# Early introduction of foods to prevent food allergy

**DOI:** 10.1186/s13223-018-0286-1

**Published:** 2018-09-12

**Authors:** Edmond S. Chan, Elissa M. Abrams, Kyla J. Hildebrand, Wade Watson

**Affiliations:** 1 Division of Allergy & Immunology, Department of Pediatrics, BC Children’s Hospital, University of British Columbia, Vancouver, BC Canada; 2 Department of Pediatrics, Section of Allergy and Clinical Immunology, University of Manitoba, Winnipeg, MB Canada; 3 Division of Allergy, Department of Pediatrics, IWK Health Centre, Dalhousie University, Halifax, NS Canada

## Abstract

Food allergy is a growing public health problem, and in many affected individuals, the food allergy begins early in life and persists as a lifelong condition (e.g., peanut allergy). Although early clinical practice guidelines recommended delaying the introduction of peanut and other allergenic foods in children, this may have in fact contributed to the dramatic increase in the prevalence of food allergy in recent decades. In January 2017, new guidelines on peanut allergy prevention were released which represented a significant paradigm shift in early food introduction. Development of these guidelines was prompted by findings from the Learning Early About Peanut Allergy study—the first randomized trial to investigate early allergen introduction as a strategy to prevent peanut allergy. This article will review and compare the new guidelines with previous guidelines on food introduction, and will also review recent evidence that has led to the paradigm shift in early food introduction.

## Background

Peanut allergy is a potentially anaphylactic food allergy which is very difficult to outgrow once acquired [1]. Although overall mortality due to peanut allergy is low, the fear of life-threatening anaphylactic reactions contributes significantly to the medical and psychosocial burden of this condition [2]. Early clinical practice guidelines recommended delaying the introduction of peanut-containing foods until the age of 3 years [3]. However, this recommendation was based on expert opinion only and likely resulted, at least in part, to the increase in peanut allergy over the last 20 years. Recently, results from the landmark Learning Early About Peanut (LEAP) study has provided Level 1 evidence (i.e., evidence from a high-quality randomized trial or prospective study) to support a paradigm shift for the early introduction of foods [4]. LEAP, which was the first randomized trial to study early allergen introduction as a preventive strategy, found that the introduction of peanut at 4–11 months of age significantly reduced the risk of developing peanut allergy in high-risk infants. Given the large number of study participants and the observed treatment effect, LEAP received extensive publicity, which resulted in the need to develop clinical practice recommendations that would help operationalize the study findings. To achieve this goal, the National Institute of Allergy and Infectious Diseases (NIAID) convened members of the Guidelines Committee and numerous other stakeholder organizations, including the Canadian Society of Allergy and Clinical Immunology (CSACI), to develop addendum guidelines on peanut allergy prevention [2]. These NIAID-sponsored guidelines represent a dramatic shift from previous advice to parents and caregivers regarding the introduction of peanut in children. This article will review and compare the new addendum guidelines with previous guidelines on food introduction, and will review recent evidence from observational studies and randomized controlled trials that has led to the paradigm shift in early food introduction. Potential challenges in implementing the new guidelines are also discussed, and key take-home messages for practitioners are provided.

## Defining an infant “at risk” of developing food allergy

Previous guidelines defined an infant at high risk of developing food allergy as one with a first-degree relative (at least one parent or sibling) with an allergic condition such as atopic dermatitis, food allergy, asthma or allergic rhinitis [5, 6]. The recent NIAID-sponsored addendum guidelines have defined “at-risk” infants very differently. According to these new guidelines, a “high risk” infant is defined as one with severe eczema and/or egg allergy, and an “at-risk” infant is defined as one with mild or moderate eczema [2]. These addendum guidelines have not included a younger sibling of a child with peanut allergy in the high-risk definition since younger siblings described in previous studies may have been at increased risk due to delayed peanut introduction [7].

How the old and new definitions of “at risk” infants should be reconciled remains to be determined, and still requires international consensus. Nonetheless, infantile eczema is increasingly being recognized as the biggest risk factor for food allergy, as per the dual allergen exposure hypothesis [8]. According to this hypothesis, cutaneous exposure to food allergens can lead to allergic sensitization, while early oral consumption of these foods may actually result in tolerance. The timing and balance of cutaneous and oral exposure determines whether a child has allergy or tolerance.

Recent randomized trials have found that moisturizing infants with a family history of atopy daily within 3 weeks of birth can prevent atopic dermatitis [9, 10] (please see *Atopic Dermatitis* article in this supplement for more information on its prevention). Investigators have hypothesized that preventing the development of atopic dermatitis through early moisturizing may also subsequently help prevent allergic sensitization to foods [10].

## When to introduce allergenic foods: old guidelines

In 2000, the American Academy of Pediatrics (AAP) recommended delaying the introduction of peanut until 3 years of age [3]. This advice was based on expert opinion rather than on prospective clinical trials, and likely contributed to the increase in the prevalence of peanut allergy in recent decades. In 2008, the AAP partially reversed the 2000 recommendation, stating that the introduction of allergenic foods “should not be delayed” [11]. However, there was insufficient data available at that time to strongly recommend that peanut “should” be introduced at approximately 6 months of age, resulting in continued confusion regarding implementation of this guideline recommendation.

Revised guidelines with regards to the introduction of allergenic foods were released in 2013 by both the American Academy of Allergy, Asthma & Immunology (AAAAI) [6] and the Canadian Paediatric Society (CPS) [5]. The CPS, for example, stated that the introduction of solid foods should not be delayed beyond 6 months, and that allergenic solids should be given regularly once introduced [5]. The CPS also discouraged routine skin prick testing (SPT) and serum immunoglobulin E (IgE) testing prior to food introduction due to false positive results that could lead to an erroneous delay in allergenic food introduction. However, these guidelines still included the somewhat ambiguous phrase “no benefit to delay”, and failed to provide parents with specific instructions on the exact age for introducing peanut-containing foods.

## Recent evidence supporting the early introduction of foods

### Observational studies

Several observational studies have suggested that early introduction of potentially allergenic foods may be associated with a decreased risk of developing food allergy. A questionnaire-based survey conducted in 2008 found that the prevalence of peanut allergy was ten-fold higher among Jewish children in the United Kingdom (UK) compared with Jewish children in Israel [12]. This difference in prevalence was attributed to earlier and more frequent peanut exposure in the first year of life in Israel compared with the UK. A population-based, cross-sectional study (HealthNuts) that included over 2500 infants found a lower risk of egg allergy among those that were introduced to egg at 4–6 month of age compared to those introduced at 10–12 months of age or later [13]. Another observational study examining the feeding history of over 13,000 infants found the incidence of IgE-mediated cow’s milk allergy to be significantly lower in infants who were introduced to cow’s milk formula within the first 14 days of life and given it regularly thereafter, compared to those who were introduced to the formula after 3 months of age [14]. Similarly, a case–control study that included approximately 200 children showed that early introduction of cow’s milk formula was associated with a lower incidence of IgE-mediated cow’s milk allergy [15]. Data from a Finnish birth cohort that included 994 children found that delaying the introduction of multiple foods, including oats (> 5 months) and wheat (> 6 months), was significantly associated with an increased risk of allergic sensitization to food and inhalant allergens [16]. Another birth cohort study conducted in the United States (US) showed that introducing solid food or cow’s milk (complementary food) at less than 4 months of age was associated with a reduced risk of peanut allergy by age 2–3 years in children with a parental history of asthma or allergy [17]. A study that included approximately 1600 children observed that delaying initial exposure to cereal grains until 6 months of age may increase the risk of developing IgE-mediated wheat allergy [18]. More recently, data from over 2100 children included in the Canadian Healthy Infant Longitudinal Development (CHILD) birth cohort study showed that delaying the introduction of cow’s milk products, egg, and peanut beyond the first year of life significantly increased the odds of sensitization to these foods [19].

### Prospective clinical trials

In recent years, randomized controlled trials have provided further support for the association between early food introduction and the prevention of food allergy. The most compelling evidence to date comes from the LEAP study, which randomized 640 high-risk infants (defined as those with severe eczema and/or egg allergy) in the UK to either early (age 4–11 months) or delayed (avoidance until age 5 years) peanut introduction. The trial showed that the early and regular (3 times per week) consumption of peanut in these high-risk infants reduced the development of peanut allergy by 86% by 5 years of age [4]. The Persistence of Oral Tolerance to Peanut extension of the LEAP study (LEAP-On) investigated whether participants who had consumed peanut in the primary trial would remain protected from peanut allergy after cessation of peanut consumption for 12 months [20]. This extension study found that the benefits of early peanut introduction persisted after 12 months of cessation of peanut consumption, supporting the concept that early peanut tolerance is not a transient phenomenon.

In the Enquiring About Tolerance (EAT) trial, 1303 exclusively breastfed infants from the general population were randomized to either early (age 3 months) or standard (age 6 months) introduction of six allergenic foods (peanut, cooked egg, cow’s milk, sesame, whitefish, and wheat) [21]. The EAT investigators hypothesized that early introduction of these allergenic foods would reduce the prevalence of food allergy by age 3 years. The intention-to-treat analysis revealed a 20% reduction in the prevalence of food allergy in the early introduction group that was not statistically significant, likely because of the high rate of non-adherence to the dietary protocol. However, in an adjusted per protocol analysis, significant reductions were seen in the rates of peanut and egg allergy in the early introduction group.

Other prospective trials have investigated the effects of early egg introduction. In the Prevention of Egg Allergy with Tiny Amount Intake (PETIT) trial, 147 Japanese infants with eczema were randomly assigned to daily consumption of heated egg powder or placebo along with aggressive treatment of eczema [22]. The study found that randomization to heated egg powder at age 6 months significantly reduced the risk of egg allergy by 78% compared with avoidance until age 12 months. The trial was stopped early due to benefit. The Solids Timing for Allergy Research (STAR) randomized 86 high-risk infants with moderate-to-severe eczema to receive pasteurized raw whole-egg powder or rice powder (placebo) at 4 months of age [23]. At 8 months, both groups were introduced to whole cooked egg under medical supervision. At 1-year, there was a non-significant trend toward a lower rate of egg allergy in the group who received pasteurized raw egg powder at age 4 months vs. whole cooked egg at age 8 months. However, the trial was terminated early due to the high rate of allergic reactions in the egg-sensitized children randomized to early introduction at age 4 months. The Starting Time of Egg Protein (STEP) study, which included 820 infants without eczema but with a family history of atopy, found that early introduction of pasteurized raw egg powder at age 4–6 months was associated with a non-significant trend toward a reduced risk of egg allergy compared to introduction at age 10 months [24]. A per-protocol analysis found that significantly fewer children in the early introduction group had IgE-mediated egg allergy at 12 months of age.

In the Beating Egg Allergy Trial (BEAT), 319 infants who were SPT-negative to egg but who had a family history of atopy were randomized to receive either pasteurized whole-egg powder or placebo at 4 months of age [25]. Subjects were treated until 8 months of age, at which time egg was introduced into the diet. At 1 year, egg sensitization was significantly lower in the treatment group compared with the placebo group. However, there was only a non-significant trend toward a reduced risk of developing egg allergy in the early introduction group. Findings from the Hen’s Egg Allergy Prevention (HEAP) study also call into question the safety of early pasteurized raw egg introduction [26]. This trial, which included 406 infants from the general population, found no evidence that early introduction of pasteurized raw egg powder at age 4–6 months prevented either egg allergy or egg sensitization. Furthermore, among the children with baseline egg sensitization who were excluded from randomization but then challenged with egg separately (n = 23), two-thirds experienced an anaphylactic reaction upon this initial introduction.

Although the results of egg allergy studies have been conflicting or inconclusive, a recent meta-analysis of randomized controlled trials investigating the timing of allergenic food introduction and the risk of developing food allergy found “moderate certainty” evidence (based on 5 trials, including 1915 children) that introducing egg between 4 and 6 months of age reduced the risk of egg allergy (relative risk [RR], 0.56; p = 0.009) [27], showing much better efficacy with using cooked as opposed to raw egg. This meta-analysis also found “moderate certainty” evidence (based on 2 trials [LEAP and EAT], 1550 patients) that peanut introduction between age 4–11 months reduced the risk of peanut allergy (RR, 0.29; p = 0.009).

## When to introduce allergenic foods: new NIAID-sponsored guidelines

**Fig. 1:**
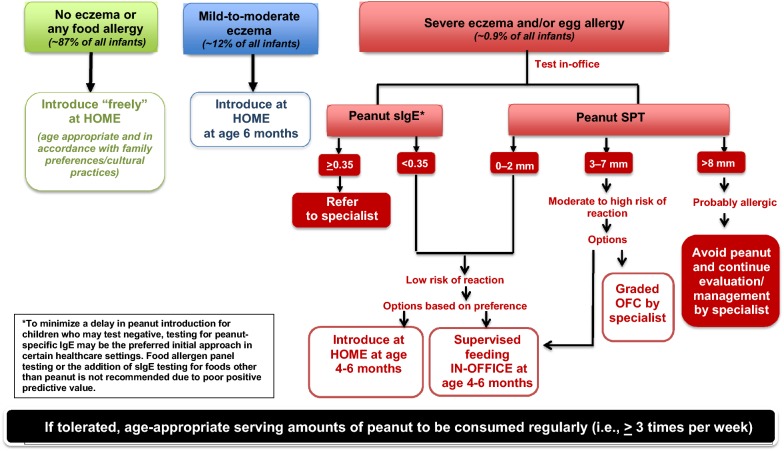
**Recommended approaches for when and where to introduce peanut and for the evaluation of children with severe eczema and/or egg allergy before peanut introduction** Adapted from: Togias et al. [2]

Given the surmounting evidence demonstrating the benefit of early allergenic food introduction—particularly the findings of the landmark LEAP study—the NIAID released addendum guidelines for the prevention of peanut allergy in January 2017 [2]. These guidelines were developed in collaboration with numerous stakeholder organizations, including the CSACI for the first time. The NIAID-sponsored addendum guidelines aimed to improve implementation by providing parents and practitioners with specific guidance on when, where and how to introduce age-appropriate peanut-containing foods. The new guidelines recommend that the highest risk infants—those with severe eczema and/or egg allergy—be introduced to age-appropriate peanut-containing food (see Table 1) as early as 4–6 months of age to reduce the risk of peanut allergy [2]. To demonstrate that the infant is developmentally ready for peanut, it is recommended that other solid foods be introduced before peanut-containing foods. For this high-risk group, allergy testing is strongly advised prior to peanut introduction. Although the preferred test is the SPT, peanut-specific IgE (sIgE) blood testing is also recommended in non-allergist settings such as family medicine, pediatrics, and dermatology, since it is more widely available. Based on these test results, either home or physician-supervised feeding is advised (see Fig. 1). Allergy tests for multiple foods other than peanut are not recommended because of their poor positive predictive value, which could lead to misinterpretation, overdiagnosis of food allergy, and unnecessary dietary restrictions [2].

**Table 1:** Typical peanut-containing foods, their peanut protein content, and feeding tips for infants [2]

	Peanut butter	Peanuts	Peanut flour or peanut butter powder	Bamba
**Amount containing approximately 2 g of peanut protein**	9–10 g or 2 teaspoons	8 g or ~ 10 whole peanuts (2½ teaspoons of grounded peanuts)	4 g or 2 teaspoons	17 g or 2/3 of a 28-g (1-oz) bag or 21 sticks
**Typical serving size**	Spread on a slice of bread or toast (16 g)	2½ teaspoons of ground peanuts (8 g)	No typical serving size	1 bag (28 g)
**Peanut protein per typical serving**	3.4 g	2.1 g	No typical serving size	3.2 g
**Feeding tips**	• For a smooth texture, mix with warm water (then let cool) or breast milk or infant formula • For older children, mix with pureed or mashed fruit or vegetables or any suitable family foods, such as yogurt or mashed potatoes	• Use blender to create a powder or paste • 2-2½ teaspoons of ground peanuts can be added to a portion of yogurt or pureed fruit or savory meal	• Mix with yogurt or applesauce	• For a smooth texture, mix with warm water (then let cool) or breast milk or infant formula and mash well • Pureed or mashed fruit or vegetables can be added • Older children can be offered sticks of Bamba

Bamba (Osem, Israel) is named because it was the product used in the LEAP trial and therefore has known peanut protein content and proven efficacy and safety. Other peanut puff products with similar peanut protein content can be substituted for Bamba

Teaspoons and tablespoons are US measures (5 and 15 mL for a level teaspoon or tablespoon, respectively)

Adapted from: Togias et al. [2]

For infants with mild-to-moderate eczema, the addendum guidelines recommend the introduction of age-appropriate peanut-containing food around 6 months of age [2]. These infants can have peanut introduced at home, without an in-office evaluation, following successful ingestion of other solid food(s). However, in-office assessment may be desired by some caregivers and providers. Among infants without eczema or any food allergy, the new guidelines state that peanut can be introduced “freely” at home, together with other solid foods and in accordance with family preferences and cultural practices.

All children who demonstrate tolerance to peanut, including those in the high-risk category, should eat peanut-containing foods regularly to maintain tolerance. As per the LEAP protocol, the guidelines advise that children consume 6–7 g of peanut protein (see Table 1 for peanut protein content of typical peanut-containing foods) per week, divided into 3 or more feedings (e.g., 2 g of protein, such as two teaspoons of peanut butter, 3 times per week). In high-risk children who have been identified as allergic to peanut following oral challenge, strict peanut avoidance and long-term evaluation and management by a specialist is advised [2] (see Fig. 1).

The CSACI has endorsed the NIAID-sponsored addendum guidelines and has recently highlighted important “take home messages” for practitioners in an editorial accompanying the guideline publication [28]. In this editorial, the CSACI emphasizes that the “overwhelming majority of infants”, including those with mild-to-moderate eczema, can be safely introduced to peanut at home, without the need for in-office investigations. The only group of infants for whom medical assessment is recommended is those with severe eczema, egg allergy or both. In this group, peanut-specific IgE testing by non-allergist physicians should only be considered when a referral to an allergist is not available in a timely manner. The CSACI also strongly discourages against the use of food panels or sIgE testing for foods beyond peanut as these could lead to misdiagnosis of food allergy and unwarranted dietary restrictions. Finally, the CSACI highlights that allergists have a “duty” to assess high-risk infants in their offices within the first year of life, and to medically supervise the first ingestion of peanut in these infants when required.

## Potential challenges in implementing the new addendum guidelines

Although the new addendum recommendations represent a major advance in the field of food allergy prevention, several experts have expressed concerns with respect to the feasibility and implementation of these guidelines [28–31]. Firstly, the prevalence of severe eczema is much lower than what most parents and practitioners believe it to be. LEAP investigators have estimated the prevalence of severe eczema to be approximately 5% [32]. However, calculations based on the prevalence of eczema in the US population suggest that only 0.9% of all infants have severe eczema, with approximately 12% and 87% having mild-to-moderate eczema or no eczema, respectively [33]. Therefore, only a very small subset of infants will require in-office testing and medically supervised peanut introduction and, as such, the vast majority of infants can have peanut introduced safely at home. Misunderstanding or misinformation among both parents and clinicians could result in many infants being misclassified as being at high-risk, leading to unnecessary screening and specialist referrals and, ultimately, delayed food introduction [34, 35]. Delaying the introduction of peanut and other solid foods due to misclassification will result in a missed opportunity for food allergy prevention. The CSACI has recently circulated a survey to examine how Canadian allergists, pediatricians, and family physicians approach early peanut introduction in their patients.

According to Turner and colleagues, another potential unintended consequence of the new guidelines is “screening creep,” in which infants who are not in a high-risk category may undergo screening, specialist referrals and even oral food challenges (OFCs) due to parental anxiety or physician over cautiousness [30]. Hence, community implementation of these addendum guidelines could have major logistical, resource and cost implications. In fact, several investigators [28, 36, 37], including those that have modeled the LEAP-based screening recommendations in the Australian [38] and Irish populations [32], have expressed similar concerns and have questioned the feasibility of implementing these recommendations outside of a clinical trial setting. Using modelled data, Shaker and colleagues recently assessed the health and economic benefits of early peanut introduction in five countries [39]. The investigators concluded that a “no-screening” approach has superior health and economic benefits in terms of number of peanut allergy cases prevented, quality-adjusted life years (QALY), and total healthcare costs compared to screening and in-office peanut introduction.

Although the addendum guidelines specifically discourage testing for other foods at the time of screening for peanut allergy, there is concern that parental pressure and physician over cautiousness may also result in some infants being tested for multiple foods. This could mistakenly result in the removal of clinically tolerated foods from the child’s diet which may, in turn, lead to loss of tolerance and the development of food allergy [30].

The relevance of egg allergy as a factor to determine when to introduce peanut at home has also been questioned. According to Wood and colleagues [29], very few children are diagnosed with egg allergy at 4–6 months of age, not because it does not exist, but primarily because it is not a common component of the infant diet at this age. However, young infants are commonly diagnosed with allergy to other foods, particularly cow’s milk. To date, there is no evidence to suggest that egg allergy is more highly associated with the development of peanut allergy than is allergy to other foods, such cow’s milk [29].

In a recent review, Abrams and colleagues [31] have also highlighted that the early introduction of solid foods contradicts current World Health Organization (WHO) recommendations [40], as well as many general pediatric guidelines (including those of the CPS) [5], that recommend exclusive breastfeeding until 6 months of age. Further studies are needed to evaluate the potential implications of earlier solid food introduction on the benefits of exclusive breastfeeding, as well as total breastfeeding duration. Furthermore, whether the age of complementary food introduction as a means of allergy prevention should be 4 months or 6 months still remains to be elucidated [41].

Historically, the allergy specialty has had to contend with overuse and misinterpretation of sIgE testing, which often results in the overdiagnosis of food allergy [42, 43]. In light of this, CSACI members, as well as other allergy experts, have expressed concerns that the new NIAID guideline option for peanut-specific IgE testing (in lieu of SPT) in non-allergy healthcare settings could potentially “do more harm than good” [28, 29]. In these settings, many infants may be misdiagnosed as having peanut allergy if they are not able to access timely specialty care for further testing, as advised by the guidelines. This, in turn, could result in unnecessary delays in the introduction of peanut and possibly other foods.

The gold standard for food allergy diagnosis remains the supervised OFC. However, infant OFC protocols are very new [44], and the limited use and experience with these protocols in Canada represents a major potential rate-limiting step to the timely assessment of high-risk infants. A delay in implementing an infant OFC beyond the 4- to 11-month window negates any potential benefit of early peanut introduction. Recently, experts have emphasized the importance of conducting a supervised OFC on the same day as the SPT (or soon after) [45, 46]. Studies are needed to determine how OFCs can be made more accessible to infants and children in Canada and other countries.

Finally, the apparent “flip flop” in recommendations for early peanut introduction has confused some parents who are not aware that the new NIAID-sponsored addendum guidelines are based on much stronger evidence than older guidelines that recommended delay. Hence, many parents remain hesitant and may delay introducing peanut, as well as other solid foods, into their infants’ diet. In fact, a recent survey of approximately 2000 new or expecting caregivers of infants younger than 1 year of age found that only 31% were willing to introduce peanut before or around 6 months of age [47]. It is important for parents to understand that there have been no reported fatalities from peanut exposure in the first year of life. Also, in Israel, where no screening is implemented and where peanut is introduced early, there is virtually no peanut allergy (i.e., prevalence = 0.17%) [12]. In contrast, the prevalence of peanut allergy is more than tenfold higher (1.85%) in the UK where peanut has traditionally been avoided in the first year of life.

## Conclusions

The increase in food allergy, particularly peanut allergy, prevalence in recent decades is a major public health problem and may, in part, be due to years of recommending delayed introduction of foods based on expert opinion only. Recent findings from observational studies, randomized controlled trials, and a meta-analysis now suggest that early introduction of allergenic foods is a potentially effective strategy for combating the rising rates of food allergy. The NIAID-sponsored addendum guidelines for the prevention of peanut allergy have led to a paradigm shift in food allergy prevention. These are the first guidelines to firmly recommend that parents “**should**” introduce non-choking forms of peanut at approximately 6 months of age, rather than the non-specific “don’t delay” message from a decade ago. Also, according to these guidelines, the vast majority of infants can have peanut introduced safely at home. This will help ensure that the window of opportunity for food allergy prevention is not missed due to delays in accessing specialty care and/or in-office allergy testing.

## Key-take home messages

- The vast majority of infants should have non-choking forms of peanut introduced at home, in an age-appropriate way, at approximately 6 months of age.
  - It is likely best to also introduce other allergenic foods (e.g., dairy, egg, non-choking forms of tree nuts, etc.) without delay (also at approximately 6 months of age).
- Only a small subset of infants with severe eczema (~ 0.9%) and/or egg allergy (i.e., high-risk infants) need in-office testing, medically supervised peanut ingestion and/or an OFC.
  - If an OFC is required, it should ideally be performed on the same day (or as soon as possible) as the first visit/SPT.
- Once introduced and tolerated, it is essential that peanut-containing foods (and other allergenic foods) be eaten regularly (e.g., 3 times per week) in amounts representative of age-appropriate servings.
- Please see *Atopic Dermatitis* article in this supplement for a discussion of early moisturizing to prevent atopic dermatitis, which could potentially help prevent food allergy as well.

## Abbreviations

SPT: skin prick testing
sIgE: specific immunoglobulin E
OFC: oral food challenge
LEAP: Learning Early About Peanut study
NIAID: National Institute of Allergy and Infectious Disease
CSACI: Canadian Society of Allergy and Clinical Immunology
AAP: American Academy of Pediatrics
AAAAI: American Academy of Allergy, Asthma & Immunology
CPS: Canadian Paediatric Society
UK: United Kingdom
EAT: Enquiring about Tolerance Study
LEAP-On: 12-month extension of the LEAP study: Persistence of Oral Tolerance to Peanut
PETIT: two-step egg introduction for prevention of egg allergy in high-risk infants with eczema
STAR: Solids Timing for Allergy Research
STEP: Starting Time for Egg Protein
US: United States
CHILD: Canadian Healthy Infant Longitudinal Development
QALY: quality-adjusted life years
